# Determinants of caregiving grandparents’ physical activity and sedentary behavior: a qualitative study using focus group discussions

**DOI:** 10.1186/s11556-023-00330-7

**Published:** 2023-10-26

**Authors:** Marie Vermote, Tom Deliens, Benedicte Deforche, Eva D’Hondt

**Affiliations:** 1 Department of Movement and Sport Sciences, Vrije Universiteit Brussel, Pleinlaan 2, 1050 Brussels, Belgium; 2 Department of Public Health and Primary Care, Ghent University, C. Heymanslaan 10, 9000 Ghent, Belgium; 3 Research Foundation – Flanders (FWO), Leuvenseweg 38, 1000 Brussels, Belgium

**Keywords:** Factors, Physical activity, Sedentary behavior, Grandparents, Grandchildren, Intergenerational, Caregiving, Focus groups, Qualitative

## Abstract

**Background:**

Evidence on the factors influencing physical activity (PA) and sedentary behavior (SB) in middle-aged and older adults taking care of their grandchild(ren) is limited, even though this knowledge seems imperative when considering the unique relationship between grandparents and their grandchild(ren) as well as the rising popularity of intergenerational interventions targeting these energy-expenditure related behaviors. Therefore, this explorative qualitative study aimed to identify the determinants of PA and SB levels among Flemish caregiving grandparents in the presence of their grandchild(ren) aged between 0–5 years.

**Methods:**

Six online focus group discussions were conducted via Microsoft Teams, all of which were audio- and videotaped with permission granted by the participating grandparents. In total, nine caregiving grandfathers and 28 caregiving grandmothers (overall mean age = 60.9 ± 4.1y) participated in this study. An inductive content analysis approach was used to derive subcategories, categories and themes from the verbatim transcribed data using NVivo R1.

**Results:**

Caregiving grandparents’ levels of PA and SB were both influenced by personal determinants (e.g., physical health, grandparental perceptions and responsibilities), interpersonal determinants (e.g., characteristics of the grandchild(ren), such as age-related physical/motor development and family interaction), and environmental determinants (e.g., weather and seasonal circumstances). PA levels of caregiving grandparents were further affected by additional personal determinants (e.g., age of the grandparent, planning and location) and interpersonal determinants (e.g., characteristics of the grandchild(ren), such as new experiences of the grandchild(ren)). Additionally, some personal determinants (e.g., perception of educational value) and interpersonal determinants (e.g., characteristics of the grandchild(ren), such as age-related cognitive development and health of the grandchild(ren)) were strictly mentioned to influence caregiving grandparents’ SB.

**Conclusions:**

Acknowledging the unique relationship between grandparents and their grandchild(ren), the current study identified specific factors determining grandparents’ PA and SB levels during the provision of grandchild care. Besides, it turned out of importance to take the interplay between the different determinants into account. Especially, for those grandparents with older grandchild(ren), within the studied 0–5 years age range, more attention should be paid to grandchild characteristics as part of the interpersonal determinants when setting up interventions to improve levels of PA and SB in caregiving grandparents.

**Supplementary Information:**

The online version contains supplementary material available at 10.1186/s11556-023-00330-7.

## Background

The aging of modern society is a well-known demographic phenomenon. Older adults, defined as people aged 65 years and older [1], are growing both in number and proportion. Aging is seen as a predominant risk factor for non-communicable diseases and chronic conditions [2]. Furthermore, the incidence of high blood pressure, high blood glucose, physical inactivity and obesity expands with increasing age, together with an impairment of many physiological systems and a decline in cognitive performance [3]. Accordingly, the maintenance of functional independence, health and quality of life is crucial for middle-aged and older adults. Especially in an aging population, increasing the duration, frequency and intensity of physical activity (PA) represents an important health promotion strategy [4]. Besides, reducing sedentary behavior (SB) has been identified as an independent predictor of successful and healthy aging in middle-aged and older adults [5].

Beyond the risk for non-communicable diseases and chronic conditions, a more advanced age is also associated with typical transitions into new life phases such as retirement, loss of a spouse and/or becoming a grandparent [6, 7]. These particular life events all seem to affect one’s habitual daily structure [6, 8, 9], as well as to influence energy-expenditure related behaviors (i.e., levels of PA and SB) [8]. Less research has focused on the transition into grandparenthood, although the grandparent role is considered one of the most important roles in the life of middle-aged and older individuals [10]. Therefore, understanding the impact of such a life-course alteration in relation to healthy aging and its social and behavioral determinants is essential [1, 11]. Furthermore, this relatively young group of middle-aged and older adults may still gain a lot in overall health status through an active and non-sedentary lifestyle.

Due to increases in female labor force participation and two-income households, higher workloads and busy social lives as well as rising rates of divorces and single-parent or newly composed families [12, 13], grandparental availability within family life is increasing worldwide [14], with grandparents be(com)ing important providers of child care (in addition to formal, institutional or public child care options) [13, 15–17]. The employment status of parents with young children as well as the availability (and cost) of formal child care seem to determine the frequency of grandchild care provided by grandparents, with two-income households relying on grandparental support almost on a daily basis [18–20]. In Belgium, this corresponds to 53.2% of grandparents looking after their grandchildren with an average of 13.4 h of care provided in a typical week [13]. Most grandparents look after their grandchildren on a regular basis, in a non-residential setting, without the presence of parents, and most often provide (intensive) care to preschool aged grandchildren (up to 3 years old) [13, 15, 19]. Additionally, especially young (grand)children (aged 0–5 years) seem to require intensive care-taking activities, whilst the interaction with older (grand)children is different as they tend to be in formal schooling in most countries [10].

The relationship between grandparents and their grandchild(ren) seems to be unique, as they play important social roles in each other’s lives that often extend well beyond frequency of contact and a relationship closeness [21]. Furthermore, according to socio-ecological models, health behaviors seem to be shaped by complex interdependence between individuals and their social, physical and policy environment [22, 23], the grandparents-grandchild dyad should be considered a particular and meaningful subgroup regarding middle-aged and older adults’ health behaviors and the underlying determinants thereof. Lately, growing interest has been shown for intergenerational programming, creating an environment in which younger and older generations closely interact with each other offering benefits for both age groups at the same time [24–26]. Regardless of the program topic (e.g., being physically active together or doing other activities, such as reading together), intergenerational programs seem to improve the confidence, self-esteem and social well-being, as well as memory and cognition in the older generation [24, 27, 28]. Existing programs including health-related behaviors, such as PA, seem to improve the PA levels of the older generation [29, 30]; however, in most cases PA was merely used as part of the intervention and rarely evaluated as an outcome. Furthermore, most intergenerational programs lack clear theoretical groundings, leading to no effects and/or no behavioral change [31]. Additionally, interventions targeting grandparents who provide complementary grandchild care, which is most common in today’s (European) society, are currently limited [32]. Especially, the intensified interactions with (young) grandchildren should be seen as a window of opportunity during which people are more susceptible to enhance health, given that the grandchild becomes an integral part of the grandparent’s life, impacting grandparents’ health through changes in lifestyle, including their own levels of PA and SB. Therefore, gaining comprehensive insight into the specific determinants of PA and SB of the grandparent in view of the grandparent-grandchild interplay/dyad during occasional care and using this information to specifically tailor interventions for this target group, may increase interventions’ effectiveness [33].

The review of King & King [33] distinguished different factors on the personal (e.g., demographics, beliefs and self-efficacy levels), interpersonal (e.g., support from family, friends, neighbors and social/cultural influences), institutional (e.g., socioeconomic and cultural aspects), and environmental/policy (e.g., climate and seasonal effects) level to be possibly determining older peoples’ PA levels. In contrast, only a handful of studies have investigated determinants of SB in older adults [34]. As with PA, determinants of SB were found within different ecological levels; however, the amount and type of factors were somewhat different compared to those determining older adults’ PA. More specifically, factors on the personal (e.g., demographics, weight and health status), interpersonal (e.g., loneliness and living with other people in the neighborhood), and environmental (e.g., mode of transport, type of housing, cultural opportunities and neighborhood safety) level have been identified in the few studies focusing on SB that were included in the systematic review by Chastin et al. [34]. However, nothing was found to be reported about factors possibly determining older adults’ SB situated at the institutional level. Yet, the included studies in the latter systematic review using qualitative methods were scarce [34]. An important shortcoming of these studies was that they usually were not specifically focusing on grandparents, but on older adults in general. Absence of information on the synergy and interaction between grandparents and their grandchild(ren) considering their unique relationship, makes it thus difficult to optimize the content and effectiveness of (future) interventions.

In view of maintaining health at an older age and the creation of evidence-based future intergenerational family interventions, a framework yielding a better understanding of why and how caregiving grandparents make certain activity choices in relation to PA and SB during the provision of grandchild care is needed. Therefore, this novel study used a qualitative research design to explore which factors determine Flemish grandparents’ PA and SB in presence of young grandchild(ren) they provide care for.

## Methods

### Design and participants

In this qualitative study, focus group discussions were used for data collection. Participants were recruited through different channels, including (social) media advertisement (e.g., Facebook, X (formerly known as Twitter) and popular newspapers) and Belgian elderly movements (e.g., OKRA 55 + , Gezinsbond). Additional participants were recruited one-on-one within the local community of the researchers’ team and by sending e-mails via the university portal. Furthermore, by using a snowball sampling strategy, all eligible participants were asked to provide the contact details of an eligible peer from their personal acquaintances. Due to the latter recruitment steps, some (*n* = 3) of the participants and the primary investigator knew each other beforehand. A project specific website was developed and launched in order to create an online platform to provide the possibility to contact the research team and the ability to sign up for participation as well as to share all relevant information and news about the study.

Grandparents interested to participate in the present study were excluded when (1) being under 50 years of age, (2) not speaking the Dutch language, (3) not being able to perform independent locomotion (e.g., relying on a walking aid or wheelchair), (4) suffering from a known cognitive impairment affecting one’s memory, attention or understanding (e.g., dementia or brain injury), or (5) living in a residential care center for elderly people. Furthermore, their grandchild(ren) had to be non-residential and could be of any sex but needed to be aged between 0 and 5 years during the planned period of data collection, since providing child care is most precarious during these early childhood years characterized by a limited independence of the child [35].

Our aim was to recruit between six and ten participants per focus group [36]. For each focus group an over-recruitment of one or two participants was pursued to anticipate potential ‘no-shows’. Focus group participants were recruited until saturation of information was reached, as in qualitative research the ultimate sample size can never be predetermined [36, 37]. However, in previously conducted focus group studies investigating energy-balance related behavior during other life phases, saturation was reached at 32 to 46 participants (i.e., equaling 5 to 7 focus groups) [38–41]. Taking this into account, participants were recruited for a maximum of 8 focus groups. As mostly grandmothers volunteered to participate in this study, the number of grandfathers were divided across the planned focus group as good as possible. Besides this, participants were divided within a focus group based on their availability on the proposed dates on which focus group discussions were organized. Furthermore, a fair division of lower and higher educated participants was arranged when composing the groups. Despite the snowball sampling technique, the investigators managed to create groups in which none of the participants knew each other.

### Ethics statement

Our qualitative study protocol was approved by the Medical Ethics Committee of the local university hospital (UZ Brussel, Vrije Universiteit Brussel; B.U.N. 1432020000191). All participants agreed to take part in the study by means of an informed consent and also gave permission for their anonymized quotes to be used in research communication. All procedures were in accordance with the ethical standards of the responsible committee on human experimentation and with the Helsinki Declaration of 1975 and its later amendments.

### Procedure

This study was performed and reported in accordance with the Consolidated criteria for reporting qualitative research (COREQ) checklist (see Additional file 1) [42]. A pre-participation questionnaire was used to determine the eligibility of grandparents volunteering to participate in the study and to be able to describe the study sample. This questionnaire included questions on socio-demographics (i.e., age, sex, educational level, living situation, marital status), linguistics, walking ability, cognitive ability, perceived health, body weight (self-reported in kg) and height (self-reported in cm), alongside some questions regarding the demographics of their grandchild(ren) (i.e., age, sex, biological relatedness) as well as the amount and frequency of grandchild care provided by the grandparent completing this preceding survey.

All focus group discussions were conducted in November and December 2020. Each discussion was led by the same female moderator (MV; PhD candidate), also being the first author of this manuscript, who was trained by senior researchers (TD and BD, respectively second and third author of this manuscript) with ample experience in focus group methodology. The moderator was assisted by two observers (LVE (female) and BC (male); both Master students in Rehabilitation Sciences and Physiotherapy), who were pre-trained by the moderator, took field notes and helped guiding the discussion to enhance quality control and assurance.

In agreement with the participants and researchers, all focus groups were organized at a convenient date and time. The initial purpose of data collection of the study was to conduct the focus groups face-to-face at university facilities or at any other low(er)-threshold location. However, due to the safety measures against the COVID-19 pandemic (i.e., social contacts in groups of 10 were not allowed), the focus group discussions were held online (i.e., via Microsoft Teams). For each focus group discussion, a duration of 60 to 90 min was foreseen, including time to provide an introduction of researchers and participants, a clear explanation of the difference between PA and SB, using specific examples, and the aim of the current study. All conversations were audio- and videotaped with permission granted by all participants.

### Question guide

A semi-structured question guide (see Table 1) was developed in agreement with all members of the research team according to recommended focus group methodology, aiming to identify factors determining caregiving grandparents’ PA and SB levels, especially in the presence of their grandchild(ren). Questions were carefully developed using appropriate literature and content validity was assured by consulting experts with ample focus group experience [36], after which they were revised by all members of the research team. The question guide was tested during the first focus group. Considering that no major changes had to be made to the question guide, the results of this test session were also included in later analysis [36]. The guide consisted of several questions, including an opening question, five key questions regarding the (grandchild care related) physical and sedentary activities of the grandparents as well as the determining factors, and an ending question. Because measures limiting grandparental childcare were still in place regarding COVID-19 at the moment of data collection, all key questions were asked in relation to the period before the pandemic. This resulted in retrospective responses of how grandparents experienced grandchild care in a normal/regular circumstances. During the focus group discussions, the moderator followed the question guide, but additional side questions were asked in order to obtain more in-depth information on the topics being raised by the participants.

**Table 1:** Focus group semi-structured question guide

**Question type**	**Question**
Opening	1. What is your name; how many grandchildren do you have; what is the name, sex and age of your grandchild(ren)?
Key questions	2. If you think about the period before measures were taken against COVID-19, were you more / less physically active while caring for your grandchild(ren) compared to when not providing care for your grandchild(ren)? a. Which factors influenced your PA when (not) providing care for your grandchild(ren)?
	3. If you think about the period before measures were taken against COVID-19, were you more / less sedentary while caring for your grandchild(ren) compared to when not providing care for your grandchild(ren)? a. Which factors influenced your SB when (not) providing care for your grandchild(ren)?
	4. If you think about the period before measures were taken against COVID-19, during which activities were you mainly physically active? a. Were you mainly physically active **together with** your grandchild(ren)? Why / why not?
	5. If you think about the period before measures were taken against COVID-19, during which activities were you mainly sedentary? a. Were you mainly sedentary **together with** your grandchild(ren)? Why / why not?
	6. If you think about the period before measures were taken against COVID-19, to what extent did caring for your grandchild(ren) take priority over other activities (organized or not) in your daily life?
Closing	7. Do you have any further remarks, suggestions, additions?

*PA* physical activity, *SB* sedentary behavior

### Data analysis

SPSS Statistics 28 software was used to analyze the data obtained from the pre-participation questionnaire and to calculate descriptive statistics of the focus group sample in its entirety. All audio-/videotapes were transcribed verbatim using the NVivo R1 software. Once the data transcription was finished, the collected data were analyzed and coded by the moderator in NVivo R1. First, an inductive content analysis approach was used based on the principles of grounded theory [43], given the exploratory nature of this study and the (relative) lack of pre-existing theories and/or research in the context of the presented study. During this first step, all quotes derived from all focus group discussions were examined for recurrent instances in order to systematically identify the recurrent quotes and group them together by means of an open coding system. Secondly, the socio-ecological model of health behavior of McLeroy et al. [44] was used in order to systematically categorize and label the determinants, and to facilitate interpretation of findings, thus using a more deductive approach to finalize data analysis [45]. The moderator and the two observers independently performed the specific categorization of the conversation content into subcategories, categories and themes to maximize the reliability of data interpretations. Accordingly, (sub)categories were in turn redefined and renamed into factors. Any doubts and disagreements were discussed among the researchers involved, until consensus was reached. All data described were checked by all co-authors afterwards.

## Results

### Study sample

In view of obtaining saturation of information, a total of six online focus group discussions were held, involving four to seven participants per focus group. A total of 48 participants originally registered to participate in our qualitative study. However, two people had to be excluded as the age of their youngest grandchild exceeded 5 years at the moment of the focus group discussion. Another two people were excluded based on the fact that their first grandchild was born during the first COVID-19 lockdown, making it impossible for them to answer questions about the period before measures were taken against the pandemic. An additional six registries cancelled upon invitation of the focus group discussion and one person did not show up during the planned focus group discussion. This resulted in a final total sample size of 37 participants, situated within the age range of 54.4–70.0 years, of which 9 were male and 28 were female, divided into six focus groups. Descriptive statistics of the study sample in its entirety are shown in Table 2.

**Table 2:** Characteristics of all focus group participants (*N* = 37)

Characteristics	Mean ± SD, %,
Sex (% female)	75.7
Age (years)	60.9 ± 4.1
BMI (kg/m^2^)	25.2 ± 3.9
Underweight (%)	2.7
Normal weight (%)	48.6
Overweight (%)	37.8
Obesity (%)	10.8
Educational level (% higher educated)	59.5
Average number of grandchild(ren) (regardless of their age) per grandparent	2.7 ± 1.3
Mean age of all grandchild(ren) (years)	4.6 ± 2.9
Average number of grandchild(ren) aged ≤ 5 years per grandparent at data collection	1.9 ± 0.9
Mean age of these grandchild(ren) (years)	3.0 ± 1.6

*SD* Standard deviation*, BMI* body mass index

### Factors determining Physical Activity (PA) and Sedentary Behavior (SB) in caregiving grandparents in presence of their grandchild(ren)

**Fig. 1:**
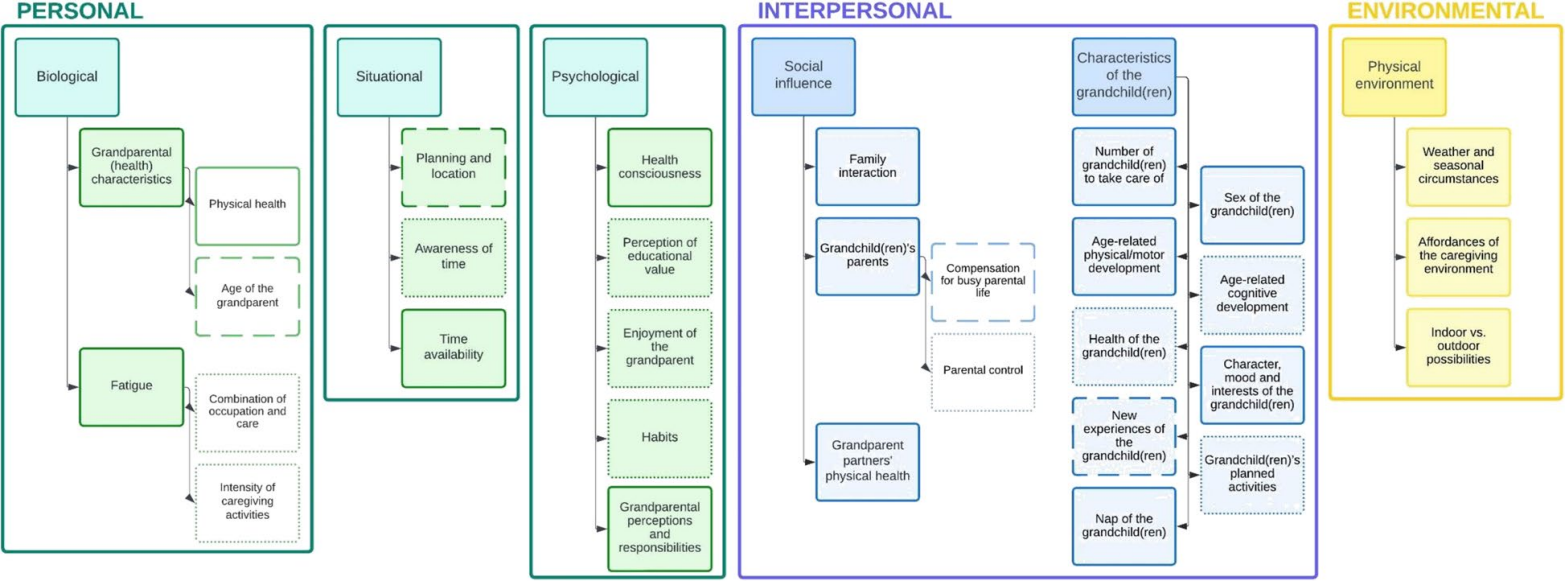
Framework of determinants influencing PA and SB in grandparents in presence of their grandchild(ren) Color-filled boxes: (sub)category; Uncolored boxes: extra subcategory; Full line: applicable for both PA and SB; Dashed line: only applicable for PA; Dotted line: only applicable for SB

An overview of the described determinants and (sub)categories determining PA and SB in grandparents when in charge of grandchild care are summarized in a framework (see Fig. 1). For each factor described below, it is indicated for which behavior it was applicable, namely PA and/or SB. Appropriate quotes for each determinant or (sub)category can be found in Tables 3, 4, 5, 6, 7 and 8.

## Personal level

### Biological

#### Grandparental (health) characteristics

##### Physical health [PA & SB]

The general health of the grandparents did play a role in the activity level of the grandparent while providing childcare. Some grandparents mentioned that when they took care of the grandchild(ren) if not feeling so well themselves, they spent more time on sedentary activities, such as playing some board games, instead of playing an active role game with the grandchild(ren) (see Table 3, Quote 1).

One grandmother had to adjust the physical activities while providing care because her physical health did not allow her to do whatever she wanted (see Table 3, Quote 2).

##### Age of the grandparent [PA only]

Grandparents’ age was also mentioned by some participants to impact on their ability to do certain physical activities while caring for the grandchild(ren). Multiple participants mentioned that the activities performed while taking care of the grandchild(ren) demanded a lot more effort with their own age increasing (see Table 3, Quote 3).

#### Fatigue

##### Combination of occupation and care [SB only]

Participants mentioned that taking care of the grandchild(ren) during the period they were still working was very exhausting. One grandmother in particular told that during the provision of care for her grandchildren, she searched for activities where she could be seated or take some extra rest in order to not overdo herself (see Table 3, Quote 4).

##### Intensity of caregiving activities [SB only]

As some physical activities were quite exhausting for the grandparents while providing grandchild care, some participants told they really needed to take some rest themselves during the period the grandchild(ren) took a nap at noon, by means of a powernap or just reading a book or newspaper while being seated (see Table 3, Quote 5).

### Situational

#### Planning and location [PA only]

It was mentioned by multiple grandparents that when the day of grandchild care and/or activities outside the home were planned in advance, this resulted in a lot more PA for themselves compared to spending the day at home (see Table 4, Quote 6).

#### Awareness of time [SB only]

When doing some specific sedentary activities, such as watching a movie, a grandparent mentioned to easily lose track of time, resulting in a mostly sedentary day of childcare for both the grandparent and grandchild(ren), with less physical activities than the grandparent had initially planned (see Table 4, Quote 7).

#### Time availability [PA & SB]

The activities which could be done with the grandchild(ren) while caring depended a lot on the time and the moment of the day the care was being provided. When the grandchild care was only provided in the evening (e.g., care after school), the activities mainly consisted of bathing the grandchild(ren), providing the grandchild(ren) with food and doing some calm (sedentary) activities, such as watching a movie together or reading a book aloud. However, when the care was provided for a whole day, more possibilities were available to go somewhere and plan multiple activities resulting in more physical activities for both parties (see Table 4, Quote 8).

### Psychological

#### Health consciousness [PA & SB]

From a health perspective, some of the participating grandparents were really strict in the activities while providing care and found it obvious to go for a walk or a bike ride when in charge of their grandchild(ren). In the same respect, some grandparents chose to not allow or to limit screentime while providing grandchild care. According to these grandparents, this resulted automatically in more physical activities for both grandparent(s) and grandchild(ren) as a replacement (see Table 5, Quotes 9&10).

#### Perception of educational value [SB only]

Most grandparents told to limit sedentary activities and for sure screentime as much as possible for their grandchild(ren) (in relation to a health conscious perspective, as mentioned above) while providing care. Yet, some of them also admitted that they allowed these kinds of activities (although limited in time) due to the educational benefits. As such, some grandparents spent a lot of time reading stories for their grandchild(ren), whereas others spent every now and then some time in front of the television together with their grandchild(ren) (see Table 5, Quote 11).

#### Enjoyment of the grandparent [SB only]

Some grandparents mentioned to engage in a lot of sedentary activities while providing grandchild care, such as reading a book aloud, listening to fairy tales or watching a movie together. They mentioned to enjoy these calm and sedentary moments together with their grandchild(ren) a lot (see Table 5, Quote 12).

#### Habits [SB only]

One grandparent in particular mentioned to want to read the newspaper every morning during breakfast, even when being in charge of grandchild care. As such, the grandchild concerned was used to the fact that every morning with the grandparents some time during breakfast had to be spent sedentary and in silence (see Table 5, Quote 13).

#### Grandparental perceptions and responsibilities [PA & SB]

Almost every grandparent mentioned that, when being in charge for grandchild care, they had the attitude of constantly being busy with the grandchild(ren) and doing every activity, either physically or sedentary, closely together with them. As such, the activity levels were completely adjusted to the needs of the grandchild(ren) (see Table 5, Quotes 14&15).

**Table 3:** Quotes about factors of grandparents’ physical activity and sedentary behavior on the personal (biological) level

Grandparental (health) characteristics	Physical health	**Quote 1** (Grandparent 25, ♀, 59 years): “If I have a pretty bad cold and I still go and look after the grandchildren, then I have a lot less energy than usual. In that case, I dare to suggest playing a board game.” **Quote 2** (Grandparent 8, ♀, 56 years): “Once we went to one of those indoor playgrounds. I was lucky that my husband was able to go with our grandchild, as I’m not that agile because I’m limited by a knee prosthesis.”
	Age of the grandparent	**Quote 3** (Grandparent 20, ♀, 61 years): “I had my first grandchild when I was 55, still working, of course you have other worries then, but physically you’re much fitter. Five years can make a difference actually. Someone told me that it is not hard to turn 50 but it is to turn 55. Now I’m 62 and the difference is noticeable.”
Fatigue	Combination of occupation and care	**Quote 4** (Grandparent 20, ♀, 61 years): “You can also let the grandchildren be physically active and at the same time find some rest for yourself. We would go to the playground or to the beach, I would sit down and rest while they played in the sand, for example, in the park or at the playground.”
	Intensity of caregiving activities	**Quote 5** (Grandparent 8, ♀, 56 years): “I also take that moment of rest in the afternoon. Because as a grandparent you need that too, to have an hour of rest. Especially with a very active grandchild, you can use that.”

♀ Quote of a grandmother

**Table 4:** Quotes about factors of grandparents’ physical activity and sedentary behavior on the personal (situational) level

Planning and location	**Quote 6** (Grandparent 37, ♂, 57 years): “We do notice that we’re much more active when the grandchildren are here. There’s a recreational area close to us, so we take them to the playground more often and then we go for a walk and sail a boat and so on. I’m not saying that we’re not active when they’re not here, but we’re less active. We do go cycling once in a while, we go for a walk once in a while, but when the grandchildren are here, we make sure that we always do some activities.”
Awareness of time	**Quote 7** (Grandparent 29, ♀, 54 years): “Once in a while, the grandchildren ask me if we’re going to watch to a fairy tale on TV, and because of that I forget the time and only after a while I notice that we’ve been sitting there for a long time.”
Time availability	**Quote 8** (Grandparent 36, ♀, 57 years): “The time grandchildren spend with the grandparents also determines a lot of the activity. If they’re only there to sleep and eat a bowl of fruit, but then go back home, you can’t go for a big walk or a trip to the playground. But if grandchildren spend the whole day with you, then you can easily organize some outdoor physical activities.”

♂ Quote of a grandfather ♀ Quote of a grandmother

**Table 5:** Quotes about factors of grandparents’ physical activity and sedentary behavior on the personal (psychological) level

Health consciousness	**Quote 9** (Grandparent 31, ♀, 58 years): “When providing grandchild care, the grandchildren can paint, they can do gymnastics, they can go on the trampoline, we go outside—which depends on the weather—but I try to fill those days with as much activity as possible.” **Quote 10** (Grandparent 31, ♀, 58 years): “When the grandchildren are with us, we want them to do other things than spend a whole afternoon watching TV, because we already have the idea that this happens a lot at home.”
Perception of educational value	**Quote 11** (Grandparent 20, ♀, 61 years): “Around 1 o'clock there’s a television show, showing live animals, such as giraffes, lions and other wildlife. The grandchildren like to watch this. Afterwards, we look for the same animals in the books again. Those are quite educational moments actually.”
Enjoyment of the grandparent	**Quote 12** (Grandparent 23, ♀, 61 years): “We go to the library together, we have a stack of 10 books and my grandson can easily sit and read books for half an hour, and then I have to read along. For me, those are real moments of rest, so he talks and reads along, and I find that delightful. Then you notice that the child also enjoys it. For me, that’s the moment that I’m really sedentary.”
Habits	**Quote 13** (Grandparent 18, ♀, 62 years): “When my granddaughter is here and she stays overnight and she’s up early in the morning, I insist to read my newspaper for half an hour or three quarters of an hour and then she has to read a book or color or do something else.”
Grandparental perceptions and responsibilities	**Quote 14** (Grandparent 30, ♂, 69 years): “When I’m with the grandchildren, we’re always physically active together. So you’re never sedentary when you’re with your grandchildren. In the playground, we play games and then our imaginations run wild and then we do all kinds of games. I’m not going to sit there on a bench and let them play. No, they always want their grandfather to be there and play with them. I don’t see many occasions where I’m not active when they are. We were doing everything together.” **Quote 15** (Grandparent 25, ♀, 59 years): “Actually, I’m always active with the grandchildren. Even when we go to the playground, my grandson always wants me to play along with him and get involved in his fantasy game. Or we’re not active together, by just playing a board game. He actually always wants to play together.”

♂ Quote of a grandfather ♀ Quote of a grandmother

## Interpersonal level

### Social influence

#### Family interaction [PA & SB]

One grandparent mentioned that the presence of the partner or the aunt of the grandchild(ren) while providing grandchild care changed the type of activities conducted with the grandchild(ren). When being alone as grandparent while taking care of the grandchild(ren), the grandparent was asked to play along with the grandchild(ren), who performed mostly physical activities. When the partner or the aunt of the grandchild(ren) was at home, more sedentary activities such as tinkering were merely chosen to be done with the grandparent, while the grandchild(ren) preferred to do the physical activities with the grandparent’s partner or the aunt being present (see Table 6, Quote 16).

Additionally, different grandparents mentioned that the help and availability of a partner made it possible to switch between more intensive physical activities with the grandchild(ren) and some less exhausting activities such as preparing lunch. Furthermore, the sometimes exhausting activities in relation to providing grandchild care could be more distributed across both grandparents.

#### Grandchild(ren)’s parents

##### Compensation for busy parental life [PA only]

A number of grandparents mentioned the fact that the parents of the grandchild(ren) were very occupied with their careers and the own household and, therefore, did not have the time to be always 100% available for their children at home and, for example, had little time to plan specific activities. As a compensation and due to the fact that grandparents probably have more free time, grandparents tend to pamper their grandchild(ren) when taking care of them by doing some extraordinary activities outside the home such as going to a theme park, visiting a zoo, and thus providing all their attention to their grandchild(ren), resulting in higher levels of PA (see Table 6, Quote 17).

##### Parental control [SB only]

Spending time in front of screens was for some grandparents not an option as activity, as this was prohibited by the parents of the grandchild(ren), pursuing a healthy lifestyle (see Table 6, Quote 18). Other parents learned their children to time and thus limit their screentime to ten minutes per occasion, resulting in less time spent sedentary by both grandparent and grandchild(ren) when grandchild care was provided by the grandparents.

#### Grandparent partners’ physical health [PA & SB]

One grandparent mentioned that the own physical activities while caring for the grandchild were limited because the partner suffered from back problems. As grandchild care was provided most of the time by both grandparents together, physical activities were limited to short walks, while the rest of the time more sedentary activities, such as making a puzzle, playing with blocks or watching some television were done with the grandchild concerned while providing care together with the partner (see Table 6, Quote 19).

### Characteristics of the grandchild(ren)

#### Number of grandchild(ren) to take care of [PA & SB]

One grandparent mentioned to like having all the grandchildren around at the same time. This resulted in more possibilities for activities and games, both active and sedentary, making it more fun for everyone. Another grandparent told that having twin grandchildren asked more PA of her compared to the care she was used to when only having a single grandchild to care of, as she had to have an extra pair of arms and eyes to keep them both calm and busy (see Table 7, Quote 20).

#### Sex of the grandchild(ren) [PA & SB]

One grandparent mentioned that when taking care of the granddaughters and giving them the possibility to choose the activities, they rather chose more sedentary and inside activities compared to when taking care of the grandsons. Although both the granddaughters and grandsons did also activities together, it was likely that the granddaughters would choose some creative activities to do inside with the grandmother, such as tinkering, while the grandfather would be more active together with the grandsons, such as playing football outside (see Table 7, Quote 21).

#### Age-related physical/motor development [PA & SB]

Overall, the age of the grandchild(ren) seemed to be an important factor for the grandparents to be more or less physically active on days of providing grandchild care. In almost all of the cases, having to take care of older grandchild(ren) meant a wider range of possible activities because of their more extensive locomotor possibilities, a wider movement area, and the fact that their repertoire of activities more and more resembled every day activities of the grandparents themselves. As younger children have a relatively small movement area, having not yet reached their maximum potential from a locomotor point of view, many grandparents mentioned that their level of PA while caring for grandchildren aged around or being younger than two years was lower compared to days without grandchild care. Yet, their level of PA seemed lower during days of providing care compared to those in absence of the grandchild(ren), because the grandchild(ren) were not yet able to perform the chosen activities (e.g., going for a walk) at the same intensity and over the same distance as the grandparent was used to. However, the grandparents of these younger grandchildren (i.e., aged around or being younger than two years) mentioned to be constantly busy with the grandchild(ren) (as they were too young to be left alone) and to feel exhausted after a day of providing care (see Table 7, Quote 22).

Furthermore, with increasing age of the grandchild(ren) also the interests of the grandchild(ren) changed. For example, one grandparent noticed that the grandchild(ren) asked more often to watch some television once they became older, resulting in more sedentary time for both the grandchild(ren) and the grandparent (see Table 7, Quote 23).

A number of grandparents mentioned that their own activity level differed on days of providing care compared to days without taking care of their grandchild(ren). Most of them were more physically active on days without grandchild care, because the daily activities of the grandparent in absence of the grandchild(ren) were too difficult to combine with providing care for the grandchild(ren) due to the child(ren)’s locomotor possibilities. Therefore, the daily activities were not executed as usual. Some grandparents tried to keep up with their daily activity schedule, such as going for a bike ride together with the grandchild(ren), although this was not possible at the same intensity as when the grandparent would have done this on his/her own (see Table 7, Quote 24).

Furthermore, some participants mentioned that the activities or movements, specifically related to taking care of young children (such as bending, lifting, sitting and playing on the floor), were quite intensive and rather uncomfortable for their age. One of these grandparents even told to experience some back pain at the end of a day of providing grandchild care (see Table 7, Quote 25).

Some of the participating grandparents had multiple grandchildren with a relative large age difference between them. These grandparents told that it was almost impossible to be physically active with all grandchildren together as the youngest ones were not able to do the activities the older ones could already conduct. This often resulted in more adjusted and sedentary activities, and thus a lower level of activity for the grandparent (see Table 7, Quote 26).

#### Age-related cognitive development [SB only]

Besides the physical/motor developmental level, children also advance in terms of their cognitive capacities with increasing age. According to the grandparents, this resulted in the ability to understand how to play board games, yielding more sedentary activities from the moment the grandchild(ren) were cognitively able to play along (see Table 7, Quote 27).

#### Health of the grandchild(ren) [SB only]

Grandparents told they were less active on days the grandchild(ren) were not feeling well, as the main activity of the grandparent was then to nurse the grandchild(ren) instead of going for a walk or a bike ride together (see Table 7, Quote 28).

#### Character, mood and interests of the grandchild(ren) [PA & SB]

Some grandparents explained that the character of each grandchild plays an enormous role in the grandparent’s level of activity during the provision of care. Some grandchildren liked to play a lot inside and on their own, resulting in a lower level of activity for the grandparent concerned, whereas others needed a lot more attention in everything they do, which may result in a higher level of activity of the grandparents involved (see Table 7, Quote 29).

Some of the grandparents intervened in choosing what activities were done while providing care at moments where the grandchild(ren) became moody. In some cases, the grandparents chose to do some physical activities such as going for a walk to see and feed ducks, for example (see Table 7, Quote 30). However, in other cases this resulted in some more sedentary time together with the grandchild(ren) by watching a movie or reading a book aloud. This was mentioned by a grandparent explaining screentime was allowed when the situation asked for it. For example, when the grandchild(ren) was/were tired and needed some time to become calm. These were moments the grandparent allowed screentime and thus spent some time sedentary together with the grandchild(ren) (see Table 7, Quote 31).

Almost all activities mentioned by the grandparents during the provision of grandchild care were based on the interest(s) of the grandchild(ren). Many grandchildren of the participating grandparents liked to read a book aloud, to watch a movie or to make a puzzle together, resulting in sedentary moments for the grandparent. However, many grandchild(ren) also liked to dance, to walk or to swing, resulting in activities which were more physically active for the caregiving grandparent (see Table 7, Quote 32).

Many of the grandparents considered the fun factor of the activities they chose to do with their grandchild(ren), so the grandchild(ren) would enjoy it and as a result be satisfied about being with their grandparents (see Table 7, Quote 33).

#### New experiences of the grandchild(ren) [PA only]

The physical activities of one grandparent in particular while providing grandchild care were physical games the grandchild(ren) learned at school or during camp(s). As a result, the grandparent was physically active a lot during such games (e.g., playing tag) (see Table 7, Quote 34).

#### Grandchild(ren)’s planned activities [SB only]

Grandparents mentioned that sometimes during the period they took care of the grandchild(ren), they were responsible for driving the grandchild(ren) around to their hobbies. Therefore, the grandparents were mainly restricted to being sedentary in their car, as multiple grandchildren had to be driven around to different after-school activities at different times of the day (see Table 7, Quote 35).

#### Nap of the grandchild(ren) [PA & SB]

The nap of young(er) grandchild(ren) was cited multiple times as a reason for some less intensive PA while providing childcare. As the grandchild(ren) had to sleep at noon, the house of the grandparent had to be calm and quiet. Because of this, most of the grandparents chose for a calm and sedentary activity themselves at the time of this nap. However, for some grandparents this was an adjustment, as they mentioned to be normally quite active during the day, without any interruption. For other grandparents, this moment of rest during the day was very welcome (see Table 7, Quote 36).

**Table 6:** Quotes about factors of grandparents’ physical activity and sedentary behavior on the interpersonal (social influence) level

	Family interaction	**Quote 16** (Grandparent 29, ♀, 54 years): “There’s a difference when I’m alone with the grandchildren or when grandpa or auntie are at home. When I’m alone with them, we do less crafts and we rather spend time on the playground, on the trampoline, on the swing or with the chickens outside. But when grandpa or auntie is at home, those physical activities are easier done with them while more crafting activities are done with me.”
Grandchild(ren)’s parents	Compensation for busy parental life	**Quote 17** (Grandparent 32, ♀, 61 years): “The two children of my eldest daughter live in a hard-working family, so I think that’s a kind of compensation behavior, that we say ‘yes, but here they can…’. We also live in a place where it’s very easy to move around a lot, so we want to make full use of that with the grandchildren, because we know that at home with their parents it’s more restricted, they’ll do it less.”
	Parental control	**Quote 18** (Grandparent 15, ♀, 66 years): “The eldest grandson wants to spend the whole day on the computer playing games, but also watching animals, watching firemen, he’s obsessed with that and his parents want to avoid this. As a solution, I try to tell him about it through children’s books.”
	Grandparent partner’s physical health	**Quote 19** (Grandparent 27, ♀, 58 years): “My husband is retired but he suffers a lot from back problems, so we can’t go for miles and miles, it’s usually a short walk.”

♀ Quote of a grandmother

**Table 7:** Quotes about factors of grandparents’ physical activity and sedentary behavior on the interpersonal (characteristics of the grandchild(ren)) level

Number of grandchild(ren) to take care of	**Quote 20** (Grandparent 33, ♀, 62 years): “The more grandchildren that are with us and who can play the board game, the more it will be played, of course. And the more fun it is.”
Sex of the grandchild(ren)	**Quote 21** (Grandparent 37, ♂, 57 years): “The difference between the grandsons and the granddaughters is striking. We have a bigger garden and then… Yes, the grandsons are more willing to come along when I say ‘let’s go and collect the eggs from the chickens’ and so on. They’re more easily involved. They help more in the garden,… And then the granddaughters will do more sedentary activities, that’s what my wife does, a bit of tinkering and reading to them and so on.”
Age-related physical/motor development	**Quote 22** (Grandparent 6, ♂, 58 years): “The way these smaller grandchildren play determines whether you’re very active or actually sedentary. Such small children, they have large play mats, they have all their toys on it and they’re always playing in the same two square meters. You sit there, you observe them, you help them, I take dozens of photos every time. So you just sit there and do nothing.” **Quote 23** (Grandparent 29, ♀, 54 years): “I do notice that as the grandchildren get older, we tend to watch movies on TV more often and that’s less active. When they’re younger, we used to go by bike and go feeding the ducks or the chickens or go on the swing, and that changes when they become older.” **Quote 24** (Grandparent 19, ♀, 69 years): “You can’t play football with little grandchildren. They’re very active but their movements are completely different. I’m used to going to the gym, to the shop, to cook, to do the grocery shopping and those are all things that you can’t do if you’re with a small grandchild like that.” **Quote 25** (Grandparent 11, ♀, 56 years): “I play more on the floor with the grandchildren, which is something I’d not normally do. That’s very pleasant, but I notice as I get older that it becomes a little more difficult.” **Quote 26** (Grandparent 34, ♀, 61 years): “We don’t actually go for many walks because the youngest grandchild is only two and a half and the oldest is nine.”
Age-related cognitive development	**Quote 27** (Grandparent 33, ♀, 62 years): “The older the grandchildren get, the more they ask to play board games. From the moment they can read and count and all those things, we noticed that these kinds of activities increase. This is also linked to more sedentary behavior for sure.”
Health of the grandchild(ren)	**Quote 28** (Grandparent 26, ♀, 61 years): “In case my grandson is ill, you don’t do much with him. Then he mainly needs his rest and then it’s more about nursing and medication and so on. You have to adapt your activities to what he needs: sleeping, providing medication, changing his diaper; so it’s physically different.”
Character, mood and interests of the grandchild(ren)	**Quote 29** (Grandparent 17, ♀, 69 years): “When my grandson is here, I have the feeling that I’ve not been sitting still all day. It really requires running after him the whole day. Of course it’s very tiring. He makes me jump on the trampoline, walking over walls, and everything. So as a grandmother, I’m always involved in his game. But the other grandchild is, like I said, totally different, and thus much calmer.” **Quote 30** (Grandparent 11, ♀, 56 years): “If my granddaughter is being a bit moody, I’ll more easily say ‘let’s go for a walk’ or we’ll go to see the ducks. Then the child’s behavior sometimes encourages me to be active, and sometimes to be sedentary.” **Quote 31** (Grandparent 9, ♂, 62 years): “The tablets are turned on when the grandchildren are tired. Then we put on the iPad and watch it together or we turn on the TV and watch it together, and we lie down in the chair together.” **Quote 32** (Grandparent 20, ♀, 61 years): “The twin grandchildren like to read books; you can consider that as a moment of rest for yourself. They walk around and go get new books. I’m also lucky that they like to dance; so I put on a CD, like K3 (which is a Flemish girls band for children). Then they both start to dance. I like to dance with them, so that’s also physical activity.” **Quote 33** (Grandparent 37, ♂, 57 years): “When the grandchildren are around, we make sure that we always do some activities, like going to the zoo, taking a day trip. Or to a bigger playground, where they can enjoy playing.”
New experiences of the grandchild(ren)	**Quote 34** (Grandparent 24, ♀, 55 years): “Many of these games, such as ‘Red light, Green light’, things like that, were learned at camp, so then you apply it at home. So indeed, you’re very active, and it is actually quite nice. The strange thing is that while you’re busy, you don’t realize it, it’s only when they’re gone in the evening that you say ‘Oh I’m tired’.”
Grandchild(ren)’s planned activities	**Quote 35** (Grandparent 33, ♀, 62 years): “Another thing that affects our ability to be physically active is the grandchildren’s activities on Wednesdays. The oldest ones already have after-school activities, they’ve sports activities and then we’re drivers for those grandchildren and that interrupts your afternoon, because they have to be there on time, they have to be dressed, you have to go and get them.”
Nap of the grandchild(ren)	**Quote 36** (Grandparent 10, ♂, 59 years): “Especially small grandchildren sleep a lot. That limits your freedom of movement. You do have the baby monitor so you can work a bit. But, for example, you can’t work in the garden in the meantime. You always have to be stand-by, so the activities are more sedentary when you have small children, who still sleep a lot.”

♂ Quote of a grandfather ♀ Quote of a grandmother

## Environmental level

### Physical environment

#### Weather and seasonal circumstances [PA & SB]

One of the factors mentioned by almost every grandparent was that the weather and season influenced the activities undertaken while providing grandchild care. In summer and with good weather, grandparents mentioned to go mainly outside with their grandchild(ren), resulting in more physical activities compared to winter and days with bad weather. During winter and on rainy days, more inside activities were done, resulting in mainly sedentary pursuits, such as board games, tinkering, or reading books (see Table 8, Quote 37).

#### Affordances of the caregiving environment [PA & SB]

A lot of participants explained they had plenty of outdoor space in their immediate home environment, which encouraged the grandchild(ren) to go outside together with the grandparents and be physically active. Having playgrounds in the neighborhood of the caregiving location also stimulated grandparents to go outside with their grandchild(ren), to go for a walk and to play some time at the playground, with grandparents mainly playing along with the grandchild(ren) or helping them to overcome obstacles (see Table 7, Quote 38).

Some of the grandparents took care of their grandchild(ren) at the house of the grandchild(ren) themselves in absence of the parents, instead of taking care of the grandchild(ren) at their own place. Because of the disappearance of daily moments of domestic work or rest periods (such as reading the newspaper), this resulted for some grandparents in more PA. Additionally, one grandparent told that the housing environment of the grandchild(ren) demanded a lot more PA of her, as the grandchild(ren) lived in a small apartment and she had to take the stairs several times a day while lifting and/or carrying the grandchild(ren) (see Table 7, Quote 39). However, another grandparent providing care at the grandchild(ren)’s home mentioned to be less physically active as the environment did not allow the grandparent to do everyday household activities he was used to do when being at his own home (see Table 7, Quote 40).

Furthermore, the presence of different toys at the caregiving location determined the PA level of both the grandchild(ren) and the grandparent who is playing along. When care was provided at the house of the grandchild(ren), lower levels of PA were reported as the grandchild(ren) tend to be less triggered by new influences (see Table 7, Quote 41).

One grandparent, who only provided grandchild care sporadically, mentioned that her home was not adjusted to receive young grandchild(ren), who might crawl a lot and still have to discover a lot. Therefore, this grandparent tried to focus as much as possible on outside activities such as cycling, going for a walk or going to the playground together when providing grandchild care (see Table 7, Quote 42).

#### Indoor vs. outdoor possibilities [PA & SB]

Some grandparents explained that activities done outside generally require more interaction with the grandchild(ren) compared to inside activities. During outside activities in the garden, for example, the grandparent was more frequently asked to play along with a game of football or to jump on the trampoline together as compared to inside activities which the grandchild(ren) could perform on their own, such as building fortresses or playing with blocks (see Table 7, Quote 43).

**Table 8:** Quotes about factors of grandparents’ physical activity and sedentary behavior on the (physical) environmental level

Weather and seasonal circumstances	**Quote 37** (Grandparent 13, ♀, 57 years): “In the winter, you’re more sedentary, even when the grandchildren come over, you just play games or something. If the weather is nice in summer, you spend more time outside, so you’re more active.”
Affordances of the caregiving environment	**Quote 38** (Grandparent 1, ♂, 59 years): “We have the advantage of having a playground two minutes away, so we can walk there. We play there for an hour, sometimes even three hours, then we come back. It’s very close. So, in that respect, we’ve become more active than before we had grandchildren.” **Quote 39** (Grandparent 25, ♀, 59 years): “Currently, the grandchildren live on the first floor in a very small apartment without an elevator. The youngest grandchild weights already 10 kilos and at the moment I find it physically very hard to carry her up the stairs. Even with an extra bag or something else you have to carry. I find that physically very heavy.” **Quote 40** (Grandparent 14, ♂, 66 years): “The middle grandchild lives with her parents in an apartment and they can hardly go outside. But she likes to go outside and sometimes it’s difficult to keep her inside. When she’s with us, we go see the horses, we go everywhere. Sometimes you don’t know where she’s heading. Then you have to make a physical effort to keep up.” **Quote 41** (Grandparent 9, ♂, 62 years): “There’s a difference between having the grandchildren in your home or having them in their home. When they’re with us, they’re constantly busy. They absolutely want to do one thing after another, they have a big toy box; one thing comes out, another thing comes out. Then we barely sit because you’re constantly doing everything together. But when we care of the grandchildren at their place, they’re a bit calmer. They have their own toys, they know what they’re doing, they sit down on the floor and do this and that. Then I dare to sit in the sofa and watch them from a distance.” **Quote 42** (Grandparent 12, ♀, 57 years): “I’m actually a plus grandmother, who jumps in for the really urgent care problems. During the holidays, of course, more than in a school year. But my house is not really set up for the grandchildren to act like they do at home. And for me that sometimes means that we’re more sedentary than when I’d have a fixed schedule to take care of my grandchildren, I think that’s also a negative impact on my physical activity level when providing care.”
Indoor vs. outdoor possibilities	**Quote 43** (Grandparent 37, ♂, 57 years): “So when we go outside, we’re active together, the grandchildren and I. On the trampoline, a bit of hopping, on the swing, the slide… The sedentary things are more inside.”

♂ Quote of a grandfather ♀ Quote of a grandmother

## Discussion

The purpose of this explorative qualitative study was to identify determinants of PA and SB levels among Flemish caregiving grandparents in the presence of their grandchild(ren) being aged between 0–5 years. Using focus group discussions, determinants could be defined at three different levels, namely the personal, interpersonal and environmental level. Some of the determinants were previously found in existing literature on PA and SB levels in the general (older) adult population. Yet, a lot of determinants found in this study were very specific and mainly linked to the interplay between grandparents and their grandchild(ren) during the provision of care. Especially the characteristics of the grandchild(ren) influencing the levels of PA and SB of the grandparents were notable. However, our findings showed that the levels of PA and SB of grandparents providing grandchild care are not influenced by one sole factor but by a combination of factors influencing each other. The specific determinants related to the caregiving grandparents’ PA and SB as well as the interactions between them will be discussed below.

At the **personal** level, grandparents participating in the current focus group study specifically mentioned biological factors such as their own physical health and age. This indicates that even if there are grandchild(ren) to take care of, some physical complaints may play a significant role in PA (dis)engagement among grandparents. Previous studies already pointed out that healthy grandparents are more likely to provide grandchild care per se [13, 46, 47]. Even though the beneficial effects of PA on health status were quoted as motivators by older adults in general [22, 23], these were not mentioned as a motivating factor to be PA by the caregiving grandparents in our study. Most motivational factors discussed during the focus group discussions were grandparent-specific and could be related to the role-model grandparents want to play in the life of their grandchild(ren) (e.g., undertaking activities together so that grandchild(ren) and grandparent are both physically active (i.e., health consciousness), always playing along with the grandchild(ren) as a grandparent (i.e., grandparental perceptions and responsibilities) or allowing the grandchild(ren) to spend some time in front of the television (i.e., perception of educational value)). Situational factors, on the personal level, were mentioned to a lesser extent to influence levels of PA or SB in the current study. Yet, most of them were related to the organization of the grandparental care (i.e., planning and location, time availability) which are factors that are not always in the power of the grandparent to be modified during the provision of grandchild care and thus probably will not provide changes in the levels of PA and SB of the grandparents while providing grandchild care.

Factors at the **interpersonal** level, and more specifically in view of the characteristics of the grandchild(ren), were mentioned most often by the participating grandparents as influencing their levels of PA and SB while providing grandchild care. The number of grandchild(ren) to take care of as well as the grandchild(ren)’s age-related development (both on the physical/motor and cognitive domains) were already reported as reasons for providing grandchild care in the first place by Aassve et al. [9] and Igel et al. [19, 48], respectively. Based on the findings of the present study, it is now clear that these factors may also impact the grandparents’ PA and SB levels when actually providing grandchild care. Besides these characteristics of the grandchild(ren) themselves, other social influences such as family interaction, the (physical health of the) partner of the grandparent as well as the parents of the grandchild(ren) (i.e., compensation for busy parental life; parental control) are considered regarding the choice of activities made by the grandparents while providing care for their grandchild(ren).

Weather and seasonal circumstances were identified as a recurring factor within the physical **environmental level **of PA and SB determinants in the current research. As the grandchild(ren) of the participating grandparents in our study were relatively young (i.e., aged between 0 and 5 years) and might be extra vulnerable for cold temperatures and rain (i.e., typical Belgian weather during winter), for example, grandparents might take this particular factor into account to a larger extent when setting up activities during the provision of grandchild care. Another common factor for the grandparents to engage in PA and limiting their SB was the availability of facilities nearby the caregiving environment. In studies focusing on older adults in general, the availability or the lack of facilities to exercise was mostly mentioned as a facilitator/barrier to be physically active, respectively [49–51]. However, the caregiving grandparents in the present study who owned a large garden or who were living close to a playground (i.e., affordances of the caregiving environment) mentioned this solely as a motivator for PA, as they were encouraged to be (more) physically active with their grandchild(ren) and limit their own SB by such facilities.

Due to their caregiving and responsible role, grandparents have a large share in their own levels of PA and SB while taking care of younger grandchildren (0–5 years) as in most cases they decide themselves what activities will be done during that time. However, just like in other research, the levels of PA and SB in grandparents while providing grandchild care gets determinant by an interplay of different determinants. Specifically in the current grandparental population, the older and more developed the grandchild(ren) get, the more the characteristics of the grandchild(ren) will influence the levels of PA and SB of the grandparent while providing care for them.

The factors found in the current study could be of use in the development of strategies aimed at increasing PA and decreasing SB among caregiving grandparents in view of maintaining health at an older age, for example, in intergenerational programs or other randomized controlled trials targeting energy-expenditure behaviors in grandparents. On the one hand, the younger the grandchildren are, the more to focus on the factors situated at the personal level, and more specifically the psychological level, such as the health consciousness of grandparents and the grandparental perceptions and responsibilities, without neglecting the social influence on the interpersonal level and the physical environmental factors such as weather and seasonal circumstances. However, the latter factors that are identified within the interpersonal and environmental level are less modifiable by the grandparents themselves. On the other hand, the older the grandchildren get, the more grandchild characteristics should be taken into account. As a result, future interventions focusing on improving grandparents’ PA and SB levels should best be tailored based on the age of the grandchild(ren). As actual PA and SB levels of young grandchildren (e.g., 0–2 years) are somewhat harder to focus on due to age-related developmental changes (e.g., both on the physical/motor and cognitive domains), interventions for caregiving grandparents with young grandchildren might be developed twofold. That is, a main focus on the actual PA and SB levels of grandparents themselves, whereas for their young grandchildren improving the level of motor competence might be the focus of interest given that in the early years it plays a key role in children’s general development as a prerequisite of PA and the pursuit of health in later life [52, 53]. Additionally, these grandparents with young grandchildren should learn to better cope with their personal determinants such as health consciousness as well as grandparental perceptions and responsibilities in order to improve levels of PA and SB. However, it should be taken in consideration that the intervention activities should be feasible to combine with the caregiving activities (i.e., mostly nurturing activities) as well as with the daily structure of the grandchild (i.e., nap of the grandchild) in order to be effective. Interventions for grandparents with older grandchildren (e.g., > 2 years) could probably be developed as intergenerational programs. If PA levels of these grandparents would need to be increased (and SB levels to be decreased), interventions could be set up focusing simultaneously on the PA of two relatives such as in the Run Daddy Run intervention [51], yet in an intergenerational context (i.e., grandparents with their grandchild(ren)), such as in the GRANDPACT project [32]. Intervention activities should focus on those pursuits that can be done together by grandparent and grandchild(ren) as the participants of the current study mentioned to spend all activities together while providing care.

Additionally, based on the determinants unraveled in the present study, it is difficult to make statements about the importance of the determinants and which should be prioritized when targeted in interventions. Hence, future quantitative research in a caregiving grandparental population investigating levels of PA and SB may want to investigate the relative importance of the determinants found in the current study, especially in light of intervention development. This may be done in analogy with previous health behavior research using quantitative scorings of identified determinants on modifiability (i.e., possibility to change the influence of the determinant in a healthful direction), relationship strength (i.e., the strength of the relation between determinant and outcome), and population-level effect (i.e., the expected impact or reach of the determinant on the targeted behavior at the population-level) [54, 55].

### Strengths and limitations

A first strength of this qualitative study is that it is the first to investigate determinants of PA and SB in caregiving grandparents. Earlier research mainly focused on determinants of PA and SB in middle-aged or older adults, but never considered grandparents taking care of their grandchild(ren) as a population of interest. Secondly, the chosen qualitative research approach by means of focus group discussions provided us with the possibility to gain better understanding of the different perspectives, expectations, motivational beliefs, experiences and activities in relation to regular or occasional grandchild caregiving, as well as to encourage people to recall as much as possible as compared to using in-depth interviews, given that the focus group approach is considered to be more naturalistic (i.e., closer to everyday conversation) and also includes dynamic group interaction. In-depth interviews could have allowed participants to control the pace and direction of the interview better than focus groups. Unlike in focus groups, participants may feel more comfortable sharing their experiences in a one-on-one setting, where they can speak freely without fear of judgment or reprisal. Yet, it should be acknowledged that by this type of interviewing some participants might have been intimidated by the group setting resulting in sharing their thoughts to a lesser extent. Another strength of this study is the use of mixed-gender focus groups. On the other hand, this can be seen as a limitation as gender-specific factors could have been raised when splitting up the focus groups by sex. Other limitations, such as the possible recall bias in the responses of the grandparents, should also be acknowledged. As already mentioned in the methods section, measures limiting grandparental childcare due to the COVID-19 pandemic were still in place at the moment of data collection. As it was the aim of the authors to get an overview of what factors influenced grandparents’ levels of PA and SB especially during the provision of grandchild care, recall questions had to be asked about the period before COVID-19 hit. It should also be noticed that the Microsoft Teams setting in which the focus group discussions were organized may have frightened some participants, as for most participating grandparents this was a new or more unusual online conversation environment. This might have caused some restraint in sharing their thoughts. Furthermore, 75% of the participants were female and the majority of the participants were higher educated, resulting in a limited generalizability of the results to the overall Flemish middle-aged and older adult providing care for grandchild(ren). However, generalizing results is not the purpose of qualitative research as it is important to get the broadest possible perspective within a topic. Additionally, a member check after the data analysis was not performed, which would have allowed us to check the validity of the findings. Another limitation was that the moderator of the focus group discussion was also the first author of the manuscript and the PI of the overarching research project. As a result, expectation and assumption bias could not be completely excluded. On the other hand, the focus group discussions were non-directed by the trained moderator and the data analysis was done separately by different researchers as earlier described in the method section. So, we do not expect this to have had a great influence on the results. Yet, it should be acknowledged that there was a rather large discrepancy between the identity (mainly age) of the moderator/principle investigator and that of the study participants. Although, this may have caused a difference in cultural capital, with possible influences on how people were willing to speak up during the focus group discussions, the discussions were very lively and comprehensive. At last, the focus groups were conducted within Flemish caregiving grandparents. Because of cultural differences in grandparental caregiving [56], the applicability of our study’s findings to other grandparental populations may be limited.

## Conclusion

The factors identified in the current study highlight the unique and complex relationship between grandparents and their grandchild(ren). Specific factors, solely applicable for grandparent-grandchild dyads, such as grandparental perceptions and responsibilities seem to determine the PA and SB levels of grandparents during grandchild care provision. Furthermore, grandchild characteristics on the interpersonal level were mentioned most frequently by the grandparents to play a significant role but this aspect seems to gain importance along with an increasing age of the grandchild(ren) within the 0–5-year-old age range. In grandparents caring for younger grandchildren future interventions should mainly focus on the psychological and situational factors to increase PA and decrease SB in view of healthy aging, without neglecting social influences (from the parents of the grandchild(ren) or other family members) and environmental factors such as weather and seasonal circumstances. As the grandchild(ren) get older, the characteristics of the grandchild(ren) should be considered to a larger extent in order to improve the levels of PA and SB in (both) grandparents (and grandchildren).

### Supplementary Information

**Additional file 1.**

## Data Availability

The datasets used and/or analyzed during the current study are available from the corresponding author upon reasonable request.

## Abbreviations

PA: Physical activity
SB: Sedentary behavior
SD: Standard Deviation
BMI: Body mass index
SPSS: Statistical Package for the Social Sciences
TV: Television
