# Supplementary material for: Determinants of caregiving grandparents’ physical activity and sedentary behavior: a qualitative study using focus group discussions
Source: Eur Rev Aging Phys Act. 2023 Oct 26;20:20. doi: 10.1186/s11556-023-00330-7 (PMC10601246; doi:10.1186/s11556-023-00330-7)
Supplement: Supplementary file 1 — Additional file 1. [file 11556_2023_330_MOESM1_ESM.docx]

# Consolidated criteria for reporting qualitative studies (COREQ): 32-item checklist

## Domain 1: Research team and reflexivity

### Personal Characteristics

1. Interviewer/facilitator

Which author/s conducted the interview or focus group?

**See page 7**

1. Credentials

What were the researcher’s credentials? E.g. PhD, MD

**See page 7**

1. Occupation

What was their occupation at the time of the study?

**See page 7**

1. Gender

Was the researcher male or female?

**See page 7**

1. Experience and training

What experience or training did the researcher have?

**See page 7**

### Relationship with participants

1. Relationship established

Was a relationship established prior to study commencement?

**See page 6**

1. Participant knowledge of the interviewer

What did the participants know about the researcher? e.g. personal goals, reasons for doing the research

**See page 8**

1. Interviewer characteristics

What characteristics were reported about the interviewer/facilitator? e.g. Bias, assumptions, reasons and interests in the research topic

**See page 29-30**

## Domain 2: study design

### Theoretical framework

1. Methodological orientation and Theory

What methodological orientation was stated to underpin the study? e.g. grounded theory, discourse analysis, ethnography, phenomenology, content analysis

**See page 9**

### Participant selection

1. Sampling

How were participants selected? e.g. purposive, convenience, consecutive, snowball

**See page 5-6**

1. Method of approach

How were participants approached? e.g. face-to-face, telephone, mail, email

**See page 5-6**

1. Sample size

How many participants were in the study?

**See page 10**

1. Non-participation

How many people refused to participate or dropped out? Reasons?

**See page 10**

### Setting

1. Setting of data collection

Where was the data collected? e.g. home, clinic, workplace

**See page 8**

1. Presence of non-participants

Was anyone else present besides the participants and researchers?

**See page 7-8**

1. Description of sample

What are the important characteristics of the sample? e.g. demographic data, date

**See page 10-11; Table 2**

### Data collection

1. Interview guide

Were questions, prompts, guides provided by the authors? Was it pilot tested?

**See page 8**

1. Repeat interviews

Were repeat interviews carried out? If yes, how many?

**No. As this was not carried out it was not reported in the manuscript**

1. Audio/visual recording

Did the research use audio or visual recording to collect the data?

**See page 8**

1. Field notes

Were field notes made during and/or after the interview or focus group?

**See page 7-8**

1. Duration

What was the duration of the interviews or focus group?

**See page 8**

1. Data saturation

Was data saturation discussed?

**See page 6-7**

1. Transcripts returned

Were transcripts returned to participants for comment and/or correction?

**See page 29**

## Domain 3: analysis and findings

### Data analysis

1. Number of data coders

How many data coders coded the data?

**See page 9**

1. Description of the coding tree

Did authors provide a description of the coding tree?

**No. As this was not provided it was not reported in the manuscript**

1. Derivation of themes

Were themes identified in advance or derived from the data?

**See page 9**

1. Software

What software, if applicable, was used to manage the data?

**See page 9**

1. Participant checking

Did participants provide feedback on the findings?

**See page 29**

### Reporting

1. Quotations presented

Were participant quotations presented to illustrate the themes / findings? Was each quotation identified? e.g. participant number

**See tables 3-9**

1. Data and findings consistent

Was there consistency between the data presented and the findings?

**See page 10, results section and tables 3-9**

1. Clarity of major themes

Were major themes clearly presented in the findings?

**See figure 1**

1. Clarity of minor themes

Is there a description of diverse cases or discussion of minor themes?

**See figure 1**
